# The Use of a Hemostasis Introducer for Percutaneous Extraction of Bile Duct Stones

**DOI:** 10.4021/gr383w

**Published:** 2012-01-20

**Authors:** Juergen Feisthammel, Micheal Moche, Joachim Mossner, Albrecht Hoffmeister

**Affiliations:** a Department of Medicine, Neurology and Dermatology, Division of Gastroenterology and Rheumatology, University of Leipzig, Liebigstrasse 20, D-04103 Leipzig, Germany; b Department of Radiology, University of Leipzig, Liebigstrasse 20, D-04103 Leipzig, Germany

**Keywords:** ERCP, PTCD, Common bile duct stones, Choledocholithiasis, Stone extraction

## Abstract

**Background:**

Choledocholithiasis is defined as presence of at least one gallstone in the bile duct. Those bile duct stones (BDS) usually are extracted by ERCP. In case the bile duct is not accessible endoscopically (e.g. after major abdominal surgery), PTCD has to be performed. Extraction of the stones via PTCD has several risks as are hemorrhage, pancreatitis and injuries of the liver tissue.

**Methods:**

We here report about our experience with a significant modification of this technique by use of a 13-french hemostasis introducer as a sheath to track the transhepatic access to the bile ducts in order to reduce time and risk.

**Results:**

Three patients were treated by use of the reported modification. In all cases, the stones were successfully removable without complications.

**Conclusion:**

We demonstrate that the use of a hemostasis introducer for percutaneous extraction of common bile duct stones seems to be promising in terms of shortening hospital stay and increasing patient safety.

## Introduction

Choledocholithiasis is defined as presence of at least one gallstone in the common bile duct. To prevent serious complications, such as cholangitis or acute biliary pancreatitis, patients with bile duct stones (BDS) should undergo ERCP. Bile duct stones detected by ERCP can usually be extracted in the same session. After performing papillotomy various techniques are available for removing impacted stones.

All endoscopic methods have in common that the bile ducts have to be accessible. However, sometimes canulation of the papilla is not successful even after use of techniques such as precut papillotomy. Furthermore, after certain surgical procedures such as Billroth-II-resection or Kausch-Whipple-resection it is often not possible to reach the papilla. Therefore, in these cases the success rate of ERCP is reported to be lower and the rate of complications is even higher [1]. Double balloon endoscopy may overcome these problems in some cases.

When ERCP for extraction of BDS can not be performed successfully, a percutaneous access to the bile ducts is mandatory. There are two possibilities, i.e. PTC as a stand-alone method or in combination with ERCP as rendezvous-technique [2-4].

Stone extraction via PTCD has been already described before with the introduction of percutaneous transhepatic papillary balloon dilatation or percutaneous transhepatic choledochoscopic lithotomy [5, 6]. Both methods have been introduced as a less invasive alternative for extraction of BDS compared with surgery. To achieve percutaneous access for the choledochoscope the bile ducts have to be punctured in a PTCD-like manner and dilatation of the access channel has to be performed for up to 14 days [7].

Once the percutaneous access has been achieved, the BDS can be extracted by pushing them forward to pass the previously dilatated papilla or by grasping them and retract them via the percutaneous access [8]. Sometimes prior fragmentation of the stone(s) is necessary due to their size. The dilatation of the papilla without direct endoscopic control has the risk of causing an unnoticeable haemorrhage, albeit this risk is reported to be very low. For balloon dilatation of the papilla an increased risk for pancreatitis has been reported [9]. Even after intraluminal fragmentation of stones there is a risk for injuring the tissue of the liver during passage with the catheters for stone extraction due to the sharp-edged stones.

We now report about a modification of this method which seems to allow percutaneous extraction of BDS without the risks described above and without the need to establish a stable percutaneus fistula. We believe that this method will be very useful in situations where the rendezvous-technique is not applicable.

## Patients and Method

Up to now we treated three patients with this method: Our first patient was an 66 years old male Caucasian, 12 years ago a Kausch-Whipple resection has been performed for therapy of carcinoma of the papilla. In 2006 choledocholithiasis has been diagnosed. BDS have been extracted via PTCD. In November 2010 he was admitted to our hospital with signs of severe cholangitis. Abdominal ultrasound showed a stone in the common bile duct and marked dilatation of the intrahepatic bile ducts. ERCP was not successful. Thus, PTCD was performed for biliary drainage. After regression of the inflammatory reaction, cholangiography showed a stone, 8 mm in diameter.

The second patient treated by the procedure we describe here was a 73-year old female patient. 2 years ago she had pylorus-preserving pancreaticoduodenectomy done for therapy of carcinoma of the pancreatic head. Because of poor tolerance she received only two cycles of adjuvant chemotherapy with gemcitabine. In February 2011 she was admitted to our hospital with septic cholangitis. Abdominal ultrasound revealed a marked dilatation of the bile ducts. PTCD had to be performed since ERCP was not successful. Cholangiography via PTC detected several stones of about 5 to 8 mm in diameter within the common bile duct.

Our third patient was a 73 year old female caucasian, 8 years ago she underwent hepaticojejunostomy after perforation of the common bile duct as complication of cholecystectomy. In 2009 a BDS was extracted via PTCD. In April 2011 she was admitted to our hospital for further evaluation of acute jaundice and fever. Her primary care provider had a MRCP done where a bile duct stone in the left hepatic duct was detectable. Due to abdominal surgery the bile ducts were not accessible via ERCP. A PTCD was performed for biliary drainage. The existence of the stone could be confirmed.

**Figure 1:**
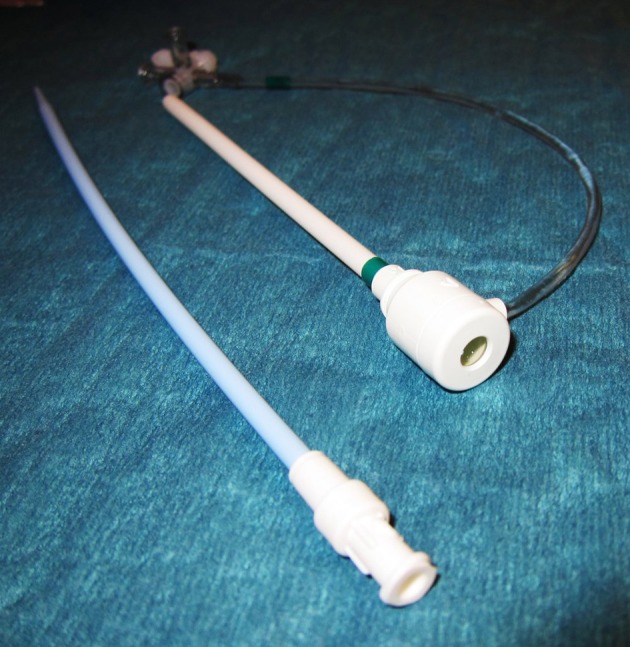
A 13-Fr Hemostasis introducer (Fast-Cath™, St. Jude Medical, Minnetonka, MN, USA) was used for easy and safe application of catheters to the bile duct over a percutaneus access.

**Figure 2:**
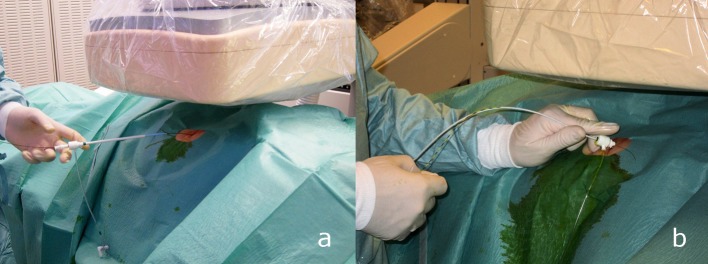
a: After having gained access to the bile duct by performing PTCD a hemostasis introducer is placed wireguided. b: The hemostasis introducer is fixed with a suture. With this access ballooncatheters and basketcatheters can be used to extract stones inside the bile ducts.

**Figure 3:**
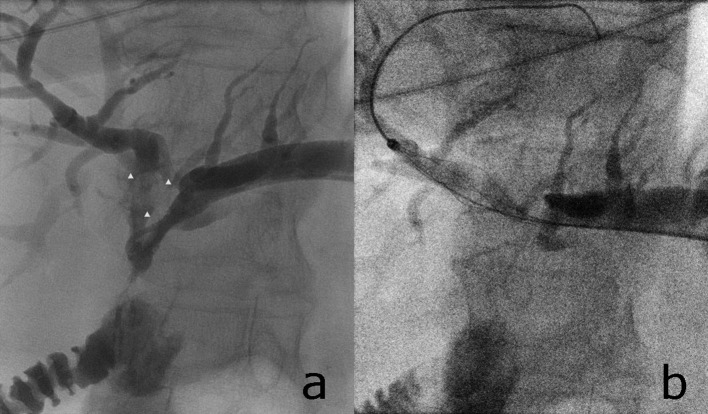
A bile duct stone is visible inside the left hepatic duct (a, white arrowheads). The stone can be grasped with a wire-guided basket-catheter (b). Access to the bile ducts is gained over a percutaneus access. A hemostasis introducer is used as described in the text.

Percutaneous transhepatic biliary drainage was performed under ultrasound guidance in the usual manner. For confirmation of existence and position of BDS a cholangiography via PTCD was performed. Next a 13-French hemostasis introducer (Fast-Cath™, St. Jude Medical, Minnetonka, MN, USA, Fig. 1) was inserted percutaneously over a guide wire (Fig. 2). The introducer was fixed by sutures. This introducer guided the application of standard catheters for stone removal, e.g. balloon catheters or wire-guided basket catheters (Fig. 3).

## Results

In our first case after PTCD and parenteral application of antibiotics the patient recovered rapidly and could be discharged. After 2 weeks the patient was readmitted for elective removal of the BDS. A hemostasis introducer was placed wire-guided over the existing PTCD. Fragmentation of all stones was possible using a wire-guided basket catheter for mechanical lithotripsy. The stone fragments were extracted, partially by pushing them forward into the duodenum using a balloon catheter, partially by grasping them with the basket catheter and retract them over the introducer. The entire procedure was possible within two sessions and a hospital stay of 5 days. After the second session PTCD was removed and the patient was discharged on the same day.

The second patient also was admitted to our hospital with severe septic cholangitis and signs of choledocholithiasis. After initial biliary drainage (PTCD) and antibiotic therapy the patient recovered. 14 days after PTCD the stones were removed in a single session using a basket catheter over a hemostasis introducer. The stones were fragmented and extracted, partially by pushing them forward into the intestine and partially by grasping and extracting over the sheath. The PTCD was flushed with saline for another 4 days to remove residual smaller fragments of the stone. At the fifth day the PTCD was removed and the patient was discharged after two more days. In our third patient, with PTCD and administration of antibiotics the clinical signs of cholangitis resolved. The bile duct stone was extracted in the same way in a single session after he recovered from cholangitis. At the end of this procedure the PTCD was removed and the patient was discharged the following day.

In all patients no serious complications such as pancreatitis, major haemorrhage, perforations or cholangitis were observed.

## Discussion

In most cases BDS can be extracted by performing ERCP. In some cases this approach is not successful due to the impossibility to canulate the papilla.

In those cases stones have to be extracted via PTCD. The stones can be removed from the common bile duct by either pushing them forward into the duodenum over a previously dilatated papilla or the stones can be extracted over the percutaneus fistula. Very often the stones have to be fragmentised prior to their extraction. Both methods have certain risks. Balloon dilatation of the papilla can cause pancreatitis. Furthermore, there is some risk for major haemorrhage. An additional problem is caused by the fact that an occurrence of bleeding can not be seen immediately due to a missing direct endoscopic view. Stone extraction over the percutaneus access after intraluminal fragmentation can cause injuries to the liver due to the often sharp-edged fragments of the stones. Therefore, it is recommended to establish a stable percutaneous fistula by dilatation over a period of up to two or three weeks.

To reduce these risks and to shorten the time of hospitalization we modified the percutaneous method by applying a 13-French hemostasis introducer as a sheath. This device tracks the distance between the bile duct and the exterior. Thus this device shields the liver tissue from the sharp-edged catheters and stones and injuries of the liver tissue are probably less likely. Furthermore, the introducer prevents collapse of the percutaneus fistula during change of catheters. As balloon dilatation of the biliary sphincter is not necessary, the risk for pancreatitis is not increased. The stones are easily to reach by wire-guided tools (e.g. baskets and balloons) without increased risks. Our experience by the use of the shortwire-system is promising. However, this method has several limitations. The introducer used by us usually is not in stock for routine ERCP everywhere. Until now, we only have limited experience with this method, albeit no complications occurred. There seems to be no additional risk compared to standard PTCD. According to our experience limitations are due to the necessity of thorough fragmentation of the stones. For larger fragments it is not possible to apply a 13 French introducer.

In summary we demonstrate that the use of a hemostasis introducer as a sheath to extract common bile duct stones percutaneously seems to be promising in terms of shortening the hospital stay and increasing patient safety.

## Abbreviations

BDS: common bile duct stones
ERCP: endoscopic retrograde cholangio-pancreaticography
MRCP: magnetic resonace cholangio-pancreaticography
PTC: Percutaneus transhepatic cholangiography
PTCD: percutaneous transhepatic cholangiodrainage.
